# EZH2 is highly expressed in pituitary adenomas and associated with proliferation

**DOI:** 10.1038/srep16965

**Published:** 2015-11-23

**Authors:** David Schult, Annett Hölsken, Sonja Siegel, Michael Buchfelder, Rudolf Fahlbusch, Ilonka Kreitschmann-Andermahr, Rolf Buslei

**Affiliations:** 1 Institute of Neuropathology, Friedrich-Alexander University Erlangen-Nürnberg (FAU), Schwabachanlage 6, 91054 Erlangen; 2 Department of Neurosurgery, Friedrich-Alexander University Erlangen-Nürnberg (FAU), Schwabachanlage 6, 91054 Erlangen; 3 Department of Neurosurgery, International Neuroscience Institute, Rudolf-Pichlmayr-Straße 4, 30625 Hannover; 4 Department of Neurosurgery, University of Duisburg-Essen, Hufelandstraße 55, 45122 Essen

## Abstract

Enhancer of zeste homolog 2 (EZH2) is a core epigenetic regulator, playing a crucial role in cell cycle regulation. The protein is known to be associated with proliferation and worse outcome in several tumor entities. In this study, we immunohistochemically investigated the expression pattern of EZH2 in a large cohort of pituitary tumors. These results were correlated with clinical features and double immunofluorescence stainings (DIS) were conducted to evaluate co-expression of EZH2 and proliferation marker Ki-67. Furthermore, we analyzed the effect of EZH2 inhibition on cell proliferation *in vitro* using the pituitary cell line AtT-20. While in the normal anterior pituitary EZH2 was almost absent, the cohort of tumors showed enhanced expression levels (p ≤ 0.0005). This was positively associated with Ki-67 indices (r = 0.834, p ≤ 0.0005) and DIF confirmed a predominant co-expression of both markers. *In vitro* experiments revealed a significant (p ≤ 0.05) decrease of tumor cell proliferation using the EZH2 inhibitor GSK126. Our results further support that epigenetic events are involved in the pathogenesis and biology of pituitary adenomas (PA). Therefore, EZH2 may function as a new potential target for therapeutic interventions in PA.

Polycomb gene (PcG) proteins were first described in drosophila melanogaster as negative regulators of homeotic genes, organizing the anterior-posterior segmentation^1^. Dependent on the context, they form large repressive complexes, polycomp repressive complex 1 and 2 (PRC1 and PRC2), which act together in epigenetic silencing of selected target genes (reviewed in^2^,^3^). Enhancer of zeste homolog 2 (EZH2) is the catalytic core protein of PRC2 and mainly known for its tri-methylation of histone H3 on lysine 27 (H3K27me3), leading to further chromatin remodeling^2^,^3^. Due to their repression of developmental genes, PRCs are of great importance in maintaining stem cell pluripotency and cell fate decision^3^. For example, knockout of EZH2 results in an increase of developmental gene expression and differentiation failures in embryonic stem cells^4^,^5^,^6^,^7^. Furthermore, PRCs retard the onset of senescence resulting in prolonged cell survival^8^. These properties make PcG proteins and especially EZH2 potential players in tumorigenesis. In the past decade, EZH2 was reported to be over-expressed in several tumor types including prostate cancer, breast cancer, lymphoma (reviewed in^9^) and brain tumors^10^. In this context EZH2 expression was often associated with poor clinical outcome and elevated proliferation rates. In addition, it has been shown that EZH2 contributes to epidermal-mesenchymal transformation and angiogenesis, leading to an increased metastatic potential of tumor cells^11^. EZH2 contributes to tumorigenesis through silencing of tumor suppressor genes^3^, but its entire function in cancer development is not yet revealed in full detail. Several observations suggest a more pleiotropic and comprehensive role of EZH2 in cancer development. For example in some myeloid disorders, missense mutations of EZH2 occur in loss of function of EZH2^12^, indicating a possible tumor suppressive role in these tumor types. This is supported by the finding that complete absence of PRC2 activity leads to stem cell death^8^. The ‘histone code hypothesis’ proposes that chromatin modulating proteins are guided to target genes through specific motifs of histone modifications^13^. EZH2 dependent tri-methylation of lysine 27 of histone H3 leads to recruitment of PRC1 and the PRC1 subunit RING1 resulting in a more condensed chromatin structure and therefore transcriptional silencing^14^,^15^. Besides chromatin interactions, EZH2 also directly controls DNA methylation through binding and recruitment of DNA methyltransferases^16^.

Considering EZH2 as a key player in tumorigenesis, several strategies have been developed focusing on inhibition of either EZH2 expression or EZH2 enzymatic activity. Small interfering RNA (siRNA) or small-molecule-S-adenosylhomocysteine hydrolase inhibitor 3-deazaneplanocin (DZNep) mediated reduction of EZH2 expression, leads to cell growth inhibition in different tumor types^17^. Whereas DZNep acts as a non-specific inhibitor of a wide range of histone methyltransferases and degrades EZH2 with subsequently inhibition of all other functions of this multifunctional protein, the very potent inhibitor GSK126 is highly selective for EZH2 methyltransferase activity^18^. It was firstly described as a potential inductor of apoptosis in various cell lines of diffuse large B cell lymphomas^18^. However, growth inhibitory effects of GSK126 were also observed in several other malignant tissues, including small cell lung cancer^19^ and prostate cancer^20^.

Pituitary adenomas (PA) are common intracranial tumors accounting for 15% of all brain neoplasms (reviewed in^21^). They represent predominantly benign lesions arising from neuroendocrine cells of the adenohypophysis (AH)^22^. Whereas well circumscribed and clinically symptomatic PA subtypes can usually be removed completely, invasive tumor growth into the cavernous sinuses, the adjacent bone structures or the brain tissue leads to incomplete neurosurgical resection and may necessitate additional radiotherapy to achieve tumor growth control. Drug-based strategies, which are standard treatment options to eliminate hormonal overproduction as well as to provide tumor control or even shrinkage in prolactin and growth hormone producing subtypes, may fail in fast recurring or atypical cases and are not sufficiently effective and established in other PA variants. In cases of regrowth after several surgical and/or radiotherapeutical interventions, treatment options are very limited and represent experimental approaches. Therefore, it is very important to establish new prognostic and therapeutic strategies dealing with fast growing and invasive PA. An important step in this direction is to understand the pathogenesis of PA which currently remains largely unknown^23^. Whereas genetic aberrations seem to be only rare events, multiple lines of evidence have described a more frequent onset of epigenetic modifications (CpG island methylation as well as histone modifications) within the tumors^24^,^25^,^26^,^27^,^28^,^29^,^30^,^31^,^32^,^33^,^34^. Therefore, and due to the important role of EZH2 in epigenetic regulation and the pathogenesis of multiple cancer types, it seems reasonable to suppose that EZH2 may also contribute to the development of PA. Against this background, we investigated the expression of EZH2 in a large cohort of human pituitary glands and related tumor samples. Based on these findings, we subsequently examined the effect of EZH2 inhibition through GSK126 on cell viability and proliferation capacity *in vitro*, using the murine ACTH secreting pituitary cell line AtT-20.

## Results

### Immunohistochemistry and double immunofluorescence staining

Using immunohistochemistry, we investigated the distribution pattern of EZH2 in 19 samples of AH and a large cohort of 165 human PA and carcinomas. Each specimen was reliably characterized by both clinical information and histopathological features according to the World Health Organization guidelines of tumors of the endocrine organs^35^. In normal AH samples (n = 19), EZH2 immunostaining was not detectable or at least restricted to single cells only (mean EZH2: 0.15%). On the contrary, nuclear EZH2 expression was significantly higher in the group of tumor samples (n = 165, mean EZH2: 2.76%, p ≤ 0.0005).

**Figure 1:**
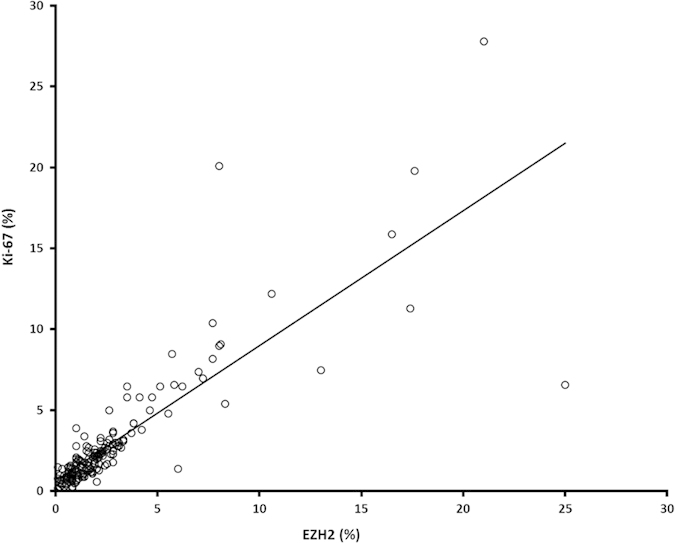
Statistical analysis revealed a distinct correlation between immunohistochemically detectable EZH2 and Ki-67 expression in pituitary adenomas (n = 165, r = 0.834, p ≤ 0.0005). The percentage of immunopositive tumor cell nuclei (a minimum of 1000 tumor cells were counted) is shown for each case (see also suppl. data).

**Figure 2:**
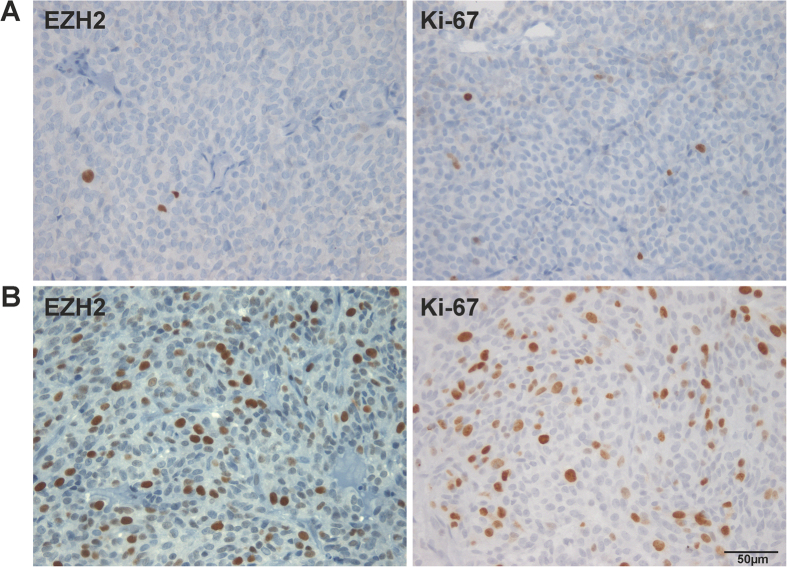
Immunohistochemical staining of EZH2 and Ki-67 in a slowly growing gonadotropin producing adenoma (**A**) and a pituitary carcinoma (**B**). The close interrelationship of elevated EZH2 expression and enhanced proliferation is clearly documented (400x).

**Figure 3:**
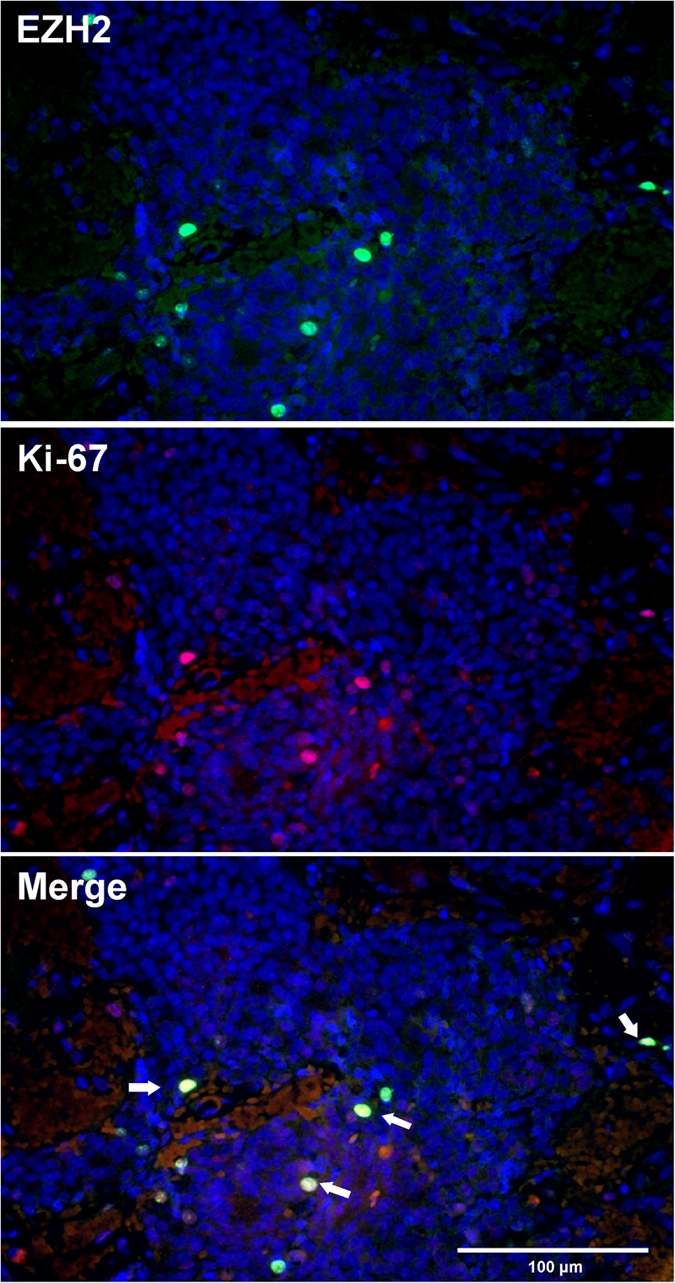
Double immunofluorescence staining of EZH2 (A, red) and Ki-67 (B, green) revealed co-localization in the majority of tumor cell nuclei (C, yellow, arrows). Nuclei (blue) were counterstained with Hoechst. Tissue: Nelson’s tumor (200x).

To evaluate a potential association between EZH2 and cell proliferation, we matched the calculated EZH2 staining rates with those of nuclear proliferation-associated antigen Ki-67. Our data revealed a distinct positive correlation of both markers (Figs 1 and 2, r = 0.834, p ≤ 0.0005. EZH2 scaled from 0% to 25%; Ki-67 scaled from 0% to 27.8%). EZH2 highly correlates with proliferation rates, irrespective of the adenoma subtype. According to this significant result, we were able to demonstrate a co-expression of both proteins in the vast majority of tumor cell nuclei, using double immunofluorescence staining (Fig. 3). We found no statistically significant association between EZH2 expression and tumor size, adenoma subtype or invasive growth pattern.

### Inhibition of EZH2 via GSK126 and its effect on H3K27me3 *in vitro*

**Figure 4:**
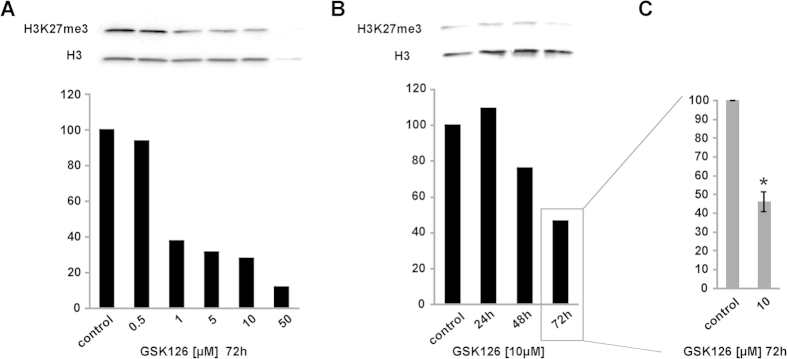
Treatment with GSK126 leads to reduced trimethylation of H3K27 in AtT-20 cells. This was shown to occur in a dose dependent manner (**A**) within 72 h. Densitometric evaluation of H3K27me3 blot is depicted below. H3 was used as loading control and served for normalization of H3K27me3 data that were compared with solvent treated AtT-20 cells set to 100%. H3K27 trimethylation decreases after 10 μM GSK126 treatment over time (**B**). Treatment with 10 μM GSK126 for 72 h was repeated in three independent experiments and densitometric evaluation showed a significant (p ≤ 0.05) decrease (**C**).

GSK126 is described to be a highly selective inhibitor of EZH2 enzyme activity and treatment leads to decreased H3K27 methylation in a wide range of malignant cell types. To prove its efficacy in PA we utilized the pituitary cell line AtT-20, and started to investigate the impact of GSK126 on tri-methylation of H3K27 (Fig. 4). Immunoblotting and densitometric analysis revealed a dose and time dependent reduction of H3K27me3 protein levels after GSK126 treatment (Fig. 4A,B). Dose escalation starting at concentrations of 0.5 μM GSK126 revealed a decreased H3K27me3 amount (Fig. 4A), being detectable after 48 h and 72 h hours (Fig. 4B). A concentration of 50 μM caused dramatic cell death and, therefore, was not taken into account. To verify the GSK126 response, treatment with 10 μM for 72 h was repeated in three independent experiments and densitometric evaluation showed a significant (p ≤ 0.05) decrease of H3K27me3 (Fig. 4C).

### Effect of GSK126 on pituitary cell viability and proliferation *in vitro*

**Figure 5:**
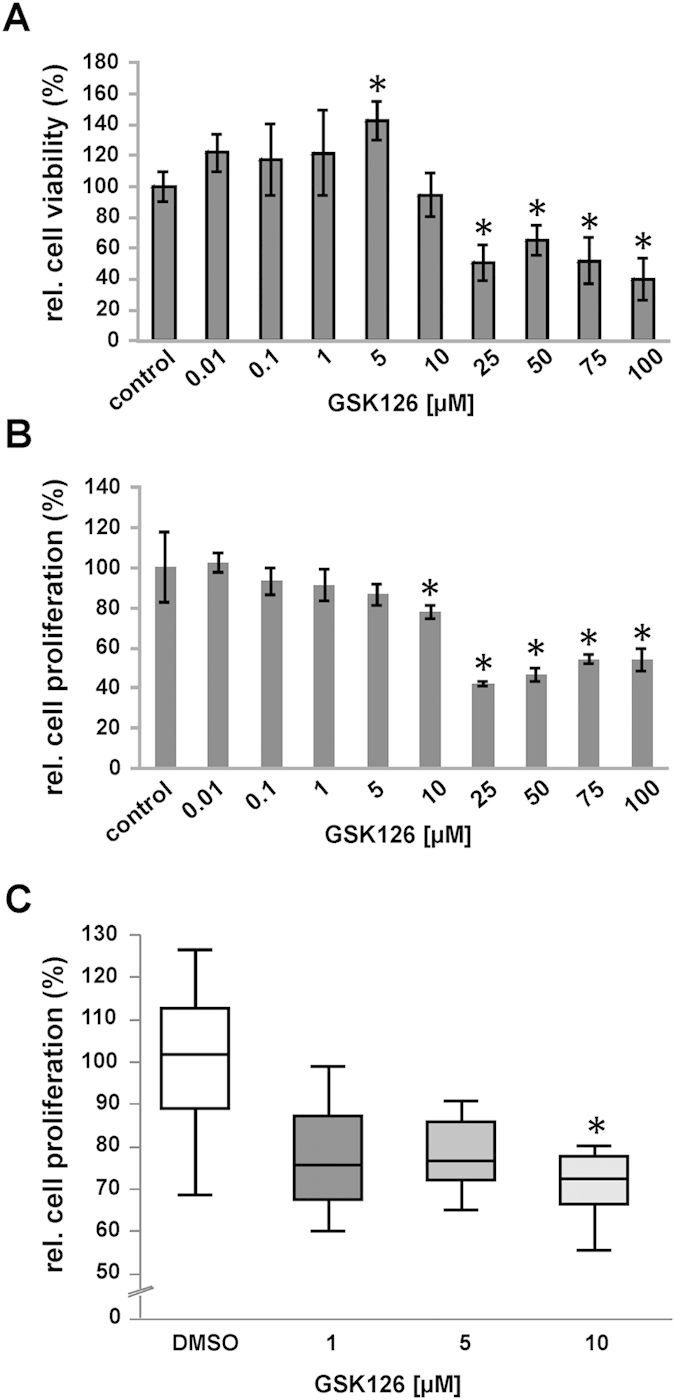
To prove a possible impact of EZH2 on cell viability and proliferation, AtT-20 cells were treated with GSK126 in a concentration ranging from 10 nM to 100 μM for 72 h. Values were compared to DMSO treated controls that were set to 100%. MTT assay revealed that cell viability decreased significantly when cells were exposed to a concentration of GSK126 >10 μM (**A**). However, BrdU assay showed that concentrations of ≥10 μM GSK126 reduced cell proliferation significantly (**B**). Two tailed paired students t-test was assessed as significant with p < 0.05. The combination of three independent BrdU examinations (**C**) confirmed a significant decrease of proliferation in AtT-20 cells when treated with 10 μM GSK126 (p < 0.05; Friedmann test with matched values and Dunn´s post test).

After showing an effective inhibition of EZH2 methyltransferase activity via GSK126 treatment *in vitro*, we examined its effect on cell viability and proliferation of AtT-20 cells. Therefore, cultured AtT-20 cells were treated with different concentrations of GSK126 and MTT viability tests (Fig. 5A) as well as BrdU proliferation assays (Fig. 5B) were performed. Values were compared to DMSO treated controls that were set to 100%. At doses above 10 μM GSK126, the amount of vital AtT-20 cells decreased significantly (p ≤ 0.05) compared to DMSO standard. However, at lower concentrations relative cell viabilities slightly increased (Fig. 5A). BrdU incorporation assays showed that treatment with concentrations of ≥10 μM GSK126, significantly decreased cell proliferation rates (Fig. 5B). We were able to confirm this result in three independent experiments (p ≤ 0.05) as shown in Fig. 5C, illustrating all relative BrdU data (n = 18) obtained from DMSO in relation to GSK126 (1 μM, 5 μM and 10 μM) treated cells.

## Discussion

The presented study is the first to demonstrate the distribution pattern and impact of the key epigenetic regulator EZH2 in a large cohort of human pituitaries and related tumors. Our results illustrate a distinct overexpression of EZH2 in the tumors in comparison to normal AH tissue and a significant correlation of this overexpression with proliferation, irrespective of the adenoma subtype. This indicates a possible role of EZH2 in both, the pathogenesis and growth progression of PA in general, which is in line with several other mainly malignant tumor entities (reviewed in^9^). Whereas PA represent a heterogeneous group of endocrine tumors, it has already been shown that the majority share the same epigenetic alterations (reviewed in^36^). For example, the EGF containing fibulin-like extracellular matrix protein 1 (EFEMP1) gene has been shown to be reduced due to inappropriate methylation in the majority of PA, irrespective of the tumor subtype^37^,^38^. Furthermore, the vast majority of PA show significantly reduced expression of microRNAs, which are known as epigenetic regulators in multiple neoplasms, leading to increased tumorigenesis^39^. Interestingly, Kitchen *et al.* found increased H3K27me3 level to be associated with silencing of exactly these microRNAs in PA, irrespective of the endocrine subtype^40^. Last but not least, hyper-acetylation of histone tails has already been associated with Ki-67 labeling index in both, typical and atypical PA^34^. Collectively these reports and our results support the assumption, that epigenetic mechanisms are involved in the pathogenesis of PA.

Furthermore, we were able to show that inhibition of EZH2 leads to decreased growth and cell viability of AtT-20 cells, suggesting a potentially new target for drug treatment in corticotropic adenomas. The very potent inhibitor GSK126 is highly selective for EZH2 methyltransferase activity and was firstly described as a potential inductor of apoptosis in various cell lines of diffuse large B cell lymphomas^18^. However, growth inhibitory effects of GSK126 were also observed in several other malignant tissues, including small cell lung cancer^19^ and prostate cancer^20^. GSK126 is competitive to S-adenosylmethionine (SAM) with a high affinity to the enzymatic activity of EZH2, resulting in overall reduced H3K27me3 levels without EZH2 degradation^18^. Based on the publication of McCabe *et al.*, several different clinical trials are currently recruiting patients with B cell lymphomas specifically for EZH2 focused treatment. Consistent with the results of McCabe *et al.*, GSK126 treatment results in both, cytostatic and cytotoxic responses of pituitary tumor cells *in vitro*. Application of ≥10 μM GSK126 for 72 h significantly inhibits the growth of AtT-20 cells. Slightly higher concentrations lead to a significantly decreased number of vital cells. Several other experimental studies have already shown that the effective concentration and duration of GSK126 treatment greatly differs depending on the tumor subtype. One cell line of a diffuse large B cell lymphoma responded to very low GSK126 doses (growth IC_50_: 28–861 nM) within two days of treatment^18^, whereas examples of Burkitt lymphoma and Hodgkin’s lymphoma as well as the B cell lymphoma cell line WILL-2 were generally less sensitive (growth IC_50_: up to >27 μM)^18^. However, the concentration of 10 μM GSK126 required to effectively suppress cellular growth of AtT-20 cells is in line with doses described for other solid tumors. Thereby, inhibition of tumor cell growth was observed in concentrations ranging from 8 μM in small cell lung cancer^19^ up to 16 μM in prostate cancer^20^. Nevertheless, for humans such doses seem rather high and side effects have to be precisely evaluated in additional *in vivo* studies. In that context, it has to be mentioned that AtT-20 cells show a modest proliferation capacity, reflecting the vast majority of human PA cases with only low Ki-67 and respective EZH2 levels. Therefore, it seems to be reasonable to assume that mainly pituitary tumors with elevated proliferation rates and high EZH2 expression (e.g. atypical PA and pituitary carcinomas) may benefit from its inhibition. The usage of AtT-20 cells for *in vitro* experiments is artificial but a sound principle that was already applied in multiple other studies^41^,^38^,^38^,^42^. Human primary adenoma cell cultures are extremely slowly growing and harbor the specific risk of fibroblast overgrowth. Furthermore, our staining results suggest EZH2 playing a role in all PA subtypes irrespective of their hormonal profile. Therefore, we decided to use this *in vitro* model recognizing that the results achieved may not be adaptable for all human PA subtypes.

Various reports have focused on the question how EZH2 is able to regulate proliferation and cancerogenesis. Although target genes of EZH2 in PA have yet to be discovered, there is multiple evidence for the possibility that epigenetic gene regulation may be involved in this process. EZH2 contributes to proliferation acting as a downstream target of the retinoblastoma (Rb) protein pathway (pRb-E2F pathway), known to be crucial in cancer development^43^,^44^. Consistent with this finding, inactivation of Rb in mice leads to development of PA^45^. Interestingly, in human pituitary tumors genetic alterations of Rb are only observed in rare pituitary carcinomas; instead, in PA the Rb promoter is highly methylated, resulting in subsequent gene silencing (reviewed in^31^).

EZH2 is needed for both, entry into and acceleration of the S phase of the cell cycle^44^. In EZH2 knockout cells, positive regulators of proliferation (e.g. cyclins) are significantly reduced, suggesting that EZH2 plays a fundamental role in the expression of these regulative genes^44^. Furthermore, EZH2 is highly expressed in epidermal progenitor cells where it controls proliferation via repression of the tumor suppressors INK4A/B, which itself act as inhibitors of cycline dependent kinases (CDK)^46^. In PA, cyclins, CDK and CDK inhibitors are implicated in the regulation of cell proliferation as well (reviewed in^31^). For example, mice lacking the CDK inhibitor p27kip1 develop PA^47^ and mice lacking the CDK inhibitor p18ink4c exhibit pituitary hyperplasia with oncogenic potential^48^. Although the gene coding for p27kip1 seems not to be mutated in PA^26^,^31^, the protein level is significantly reduced^49^,^50^, suggesting epigenetic modifications. Interestingly, in pancreatic cancer, p27kip1 acts as a target of EZH2 and EZH2 induced silencing leads to increased proliferation and malignancy^51^. Despite our large sample size, in our study we were not able to show a significant association between EZH2 rates and clinical tumor behavior, especially size and invasiveness, possibly because of the low proportion of tumor samples with a high proliferative capacity. Therefore, further clinical studies especially with those tumors that are fast recurrent and refractory to standard care are mandatory to address a potential prognostic impact of EZH2 in PA.

EZH2 is associated with tumor cell proliferation in PA and its specific inhibitor GSK126 is able to affect tumor cell viability and proliferation *in vitro*. Our results suggest a potential role of EZH2 as a target for new drug-based treatment strategies in highly proliferating PA, especially corticotropic adenoma subtypes. Further studies are required to unravel potential EZH2 targets in pituitary tumors and to analyze its prognostic and therapeutic impact.

## Methods

### Patient subjects and tissue samples for immunohistochemical investigation

A total of 184 surgical specimens of patients with sellar exploration were selected retrospectively from the archive of the Department of Neuropathology at the University Hospital of Erlangen (compare supplemental data). All patients had been surgically treated at the Departments of Neurosurgery, University Hospital Erlangen and International Neuroscience Institute (INI), Hannover. Normal AH tissue (n = 19) was collected from patients with sellar exploration (n = 18) and from one patient with Rathke’s cleft cyst (n = 1). We selected only cases with sufficient tumor material and relevant clinical data being available, without any other preselection. AH samples showed typically regular reticulin fiber network and a physiological spectrum of hormone expression. All tumor samples were clearly characterized according to the currently valid version of the World Health Organization classification of tumors of endocrine organs^35^ using hematoxylin-eosin (HE) staining as well as immunhistochemical hormone profiling. The tumor group (n = 165) consists of a large cohort of PA (n = 163) and two additional cases of pituitary carcinomas. Data on tumor size and invasive growth was carefully reviewed under inclusion of surgical reports, radiological images and histological parameters, irrespective of the affected anatomical structures. The entire collective consists of specimens of 101 male and 83 female patients with an age ranging from 13 to 87 years (mean age 51 years). Patients included in the study had given their informed consent for processing the specimens.

### Immunohistochemistry and double immunofluorescence staining

Immunhistochemical analysis was performed as described previously^52^. We used antibodies against EZH2 (1:100, rabbit, clone D2C9, Cell Signaling, Danvers, USA) and anti-Ki67 (1:200, rabbit, clone SP6, Cell Marque, Rocklin, USA). Antibodies against AH hormones were used for hormone profiling and tumor classification^52^. Double immunofluorescence staining was performed as shown previously^53^ using the above mentioned EZH2 antibody and Ki-67 (1:200, mouse, clone K-2, Zytomed, Berlin, Germany). Positive and negative controls were used to validate the staining.

### Statistical analysis of immunohistochemical investigation

For statistical analysis we used GraphPad Prism 4 for Windows (GraphPad Software Version 4.02, San Diego, California, USA) and Microsoft Excel 2010 (Microsoft, Redmond, USA). Immunostaining of EZH2 and Ki-67 was analyzed without knowledge of the diagnosis or any clinical information. From each slide at least 1000 cells, located in the particular region of main expression, were counted and percentage of positive nuclear staining was recorded. Correlation analysis was established using Pearson’s correlation coefficient (r). To identify possible differences in the EZH2 expression between the control group (normal AH) and the tumor group, as well as in the groups of invasive and non-invasive tumor samples, we used a Wilcoxon rank-sum test and compared the arithmetic average and median of EZH2 distribution in the groups, respectively. To establish a possible correlation of its expression and tumor size, we compared EZH2 levels with the largest diameter measured by the neurosurgeons and in high resolution MR images. A p-value of ≤0.05% was considered as statistical significant. We did not perform stereological quantification.

### Cell culture

The murine pituitary adrenocorticotropin (ACTH) secreting AtT-20 cell line (ATTC®-CCL-89™, LGC-Standards GmbH, Wesel, Germany) was cultured as recommended by ATCC using F-12K Medium (ATCC® 30-2004™, Manassas, USA), supplemented with Fetal Bovine Serum (5%, Biochrom, Berlin, Germany) and Horse Serum (15%, Biochrom) at 37 ^o^C and 5% CO_2_ atmosphere. Cells were treated with the highly selective EZH2 inhibitor GSK126 (Cayman Chemical Company, Ann Arbor, USA) dissolved in DMSO (Sigma-Aldrich, Steinheim, Germany) in a dose and time dependent manner, ranging from 10 nM to 100 μM and 24 to 72 hours.

### Protein preparation and immunoblotting

For Histone protein extraction we used the Qproteome™ Nuclear Protein Kit (Qiagen, Hilden, Germany) following manufactures recommendations. Protein concentration was measured using the Qubit® Protein Assay Kit (Life Technologies, Oregon, USA). SDS page and immunoblotting were performed as described previously^54^. To prove the effectivity of GSK126 treatment, membranes were incubated with Anti-trimethyl-Histone H3 (Lys27) polyclonal rabbit antibody (1:5000, Millipore, Temecula, USA). Equal protein load (10μg/lane) was estimated using anti-histone H3, CT, pan antibody of rabbit antiserum (1:50.000, Upstate, Temecula, USA) on the same membrane after membrane stripping. A volume of 200 ml stripping buffer is composed of 1.88 g glycine (Carl Roth GmbH, Karlsruhe, Germany), 7.3 g NaCl (Roth), 2.5 ml Tween20 (Sigma-Aldrich) and 14 μl β-Mercaptoethanol (Sigma-Aldrich) dissolved in deionized water. Primary antibodies were identified using HRP linked goat anti rabbit secondary antibodies (1:5000, Bio-Rad, Hercules, USA). Protein detection was facilitated by the Clarity^TM^Western ECL substrate kit (Bio-Rad). For densitometic quantification the quantitiy of every band was estimated as area under its intensity profile curve using the Quantity one® software Version 4.6.7 (Bio-Rad). To compare the amount of tri-methylated lysine 27 of histone 3 (H3K27me3) in relation to DMSO treated control (set to 100%) data of each band was normalized to corresponding total H3 values. Therefore quantity of each H3K27me3 condition was corrected by the multiplication of the ratio resulting of the band quantity value of the H3 control and H3 value of the respective condition. Changes in H3K27me3 of AtT-20 cells, treated with 10 μM GSK126 for 72 h, were evaluated in three independent experiments.

### Cell viability and proliferation assays

To analyze the impact of EZH2 inhibition on cell viability and proliferation, the AtT-20 cell line was treated with varying concentrations of GSK126 for 72 h. Cell viability and proliferation was examined using MTT (3-(4,5-dimethylthiazol-2-yl)-2,5-diphenyl-tetrazolium-bromide) test (Sigma-Aldrich) and BrdU (bromodeoxyuridine) incorporation assay (Roche, Mannheim, Germany) as described elsewhere^55^, according to manufactures instructions in three independent experiments and results were explored in comparison to solvent control, set to 100%. Each treatment condition within one experiment was carried out in sextuplicates and was repeated three times in independent experiments for the GSK126 concentrations of 1 μM, 5 μM and 10 μM.

### Statistical analysis of cell culture experiments

The value of proliferation and cell viability (sextuplicates) for each separate analysis after GSK126 treatment, was calculated statistically in relation to the DMSO treated control by a paired, two tailed t-test. The impact of EZH2 inhibition using GSK126 on AtT-20 cell proliferation was estimated in three independent BrdU incorporation assays and analyzed statistically with a one-way ANOVA Friedmann test matching values of corresponding analyses and Dunn´s post-test for multiple comparisons. Statistical analysis was conducted using Microsoft Excel 2010 and GraphPad Prism 4 for Windows. Differences were considered significant with p ≤ 0.05.

**Ethical standards.** All patients had given their informed consent for processing the specimens. The study was approved by the local ethics committee of the University Erlangen. Procedures were conducted in accordance with the Declaration of Helsinki.

## Additional Information

**How to cite this article**: Schult, D. *et al.* EZH2 is highly expressed in pituitary adenomas and associated with proliferation. *Sci. Rep.* **5**, 16965; doi: 10.1038/srep16965 (2015).

## Supplementary Material

Supplementary Information

## References

[b1] Lewis E. B. A gene complex controlling segmentation in Drosophila. Nature 276, 565–570 (1978). 103000 10.1038/276565a0

[b2] Margueron R. & Reinberg D. The Polycomb complex PRC2 and its mark in life. Nature 469, 343–349, doi: 10.1038/nature09784 (2011). 21248841 PMC3760771

[b3] Sparmann A. & van Lohuizen M. Polycomb silencers control cell fate, development and cancer. Nat Rev Cancer 6, 846–856, doi: nrc1991 [pii]10.1038/nrc1991 (2006). 17060944 10.1038/nrc1991

[b4] Chamberlain S. J., Yee D. & Magnuson T. Polycomb repressive complex 2 is dispensable for maintenance of embryonic stem cell pluripotency. Stem cells 26, 1496–1505, doi: 10.1634/stemcells.2008-0102 (2008). 18403752 PMC2630378

[b5] Pasini D., Bracken A. P., Hansen J. B., Capillo M. & Helin K. The polycomb group protein Suz12 is required for embryonic stem cell differentiation. Molecular and cellular biology 27, 3769–3779, doi: 10.1128/MCB.01432-06 (2007). 17339329 PMC1899991

[b6] Shen X. *et al.* EZH1 mediates methylation on histone H3 lysine 27 and complements EZH2 in maintaining stem cell identity and executing pluripotency. Molecular cell 32, 491–502, doi: 10.1016/j.molcel.2008.10.016 (2008). 19026780 PMC2630502

[b7] van der Stoop P. *et al.* Ubiquitin E3 ligase Ring1b/Rnf2 of polycomb repressive complex 1 contributes to stable maintenance of mouse embryonic stem cells. PloS one 3, e2235, doi: 10.1371/journal.pone.0002235 (2008). 18493325 PMC2375055

[b8] Sauvageau M. & Sauvageau G. Polycomb group proteins: multi-faceted regulators of somatic stem cells and cancer. Cell stem cell 7, 299–313, doi: 10.1016/j.stem.2010.08.002 (2010). 20804967 PMC4959883

[b9] Chase A. & Cross N. C. Aberrations of EZH2 in cancer. Clin Cancer Res 17, 2613–2618, doi: 1078-0432.CCR-10-2156 [pii]10.1158/1078-0432.CCR -10–2156 (2011). 21367748 10.1158/1078-0432.CCR-10-2156

[b10] Crea F., Hurt E. M. & Farrar W. L. Clinical significance of Polycomb gene expression in brain tumors. Mol Cancer 9, 265, doi: 10.1186/1476-4598-9-265 (2010). 20920292 PMC2958980

[b11] Crea F. *et al.* EZH2 inhibition: targeting the crossroad of tumor invasion and angiogenesis. Cancer Metastasis Rev 31, 753–761, doi: 10.1007/s10555-012-9387-3 (2012). 22711031

[b12] Ernst T. *et al.* Inactivating mutations of the histone methyltransferase gene EZH2 in myeloid disorders. Nature genetics 42, 722–726, doi: 10.1038/ng.621 (2010). 20601953

[b13] Jenuwein T. & Allis C. D. Translating the histone code. Science 293, 1074–1080 (2001). 11498575 10.1126/science.1063127

[b14] Francis N. J., Kingston R. E. & Woodcock C. L. Chromatin compaction by a polycomb group protein complex. Science 306, 1574–1577, doi: 10.1126/science.1100576 (2004). 15567868

[b15] Zhou W. *et al.* Histone H2A monoubiquitination represses transcription by inhibiting RNA polymerase II transcriptional elongation. Molecular cell 29, 69–80, doi: 10.1016/j.molcel.2007.11.002 (2008). 18206970 PMC2327256

[b16] Vire E. *et al.* The Polycomb group protein EZH2 directly controls DNA methylation. Nature 439, 871–874, doi: nature04431 [pii]10.1038/nature04431 (2006). 16357870 10.1038/nature04431

[b17] Piunti A. & Pasini D. Epigenetic factors in cancer development: polycomb group proteins. Future oncology 7, 57–75, doi: 10.2217/fon.10.157 (2011). 21174538

[b18] McCabe M. T. *et al.* EZH2 inhibition as a therapeutic strategy for lymphoma with EZH2-activating mutations. Nature 492, 108–112, doi: 10.1038/nature11606 (2012). 23051747

[b19] Sato T. *et al.* PRC2 overexpression and PRC2-target gene repression relating to poorer prognosis in small cell lung cancer. Scientific reports 3, 1911, doi: 10.1038/srep01911 (2013). 23714854 PMC3665955

[b20] Takeshima H., Wakabayashi M., Hattori N., Yamashita S. & Ushijima T. Identification of coexistence of DNA methylation and H3K27me3 specifically in cancer cells as a promising target for epigenetic therapy. Carcinogenesis 36, 192–201, doi: 10.1093/carcin/bgu238 (2015). 25477340

[b21] Daly A. F., Tichomirowa M. A. & Beckers A. The epidemiology and genetics of pituitary adenomas. Best practice & research. Clinical endocrinology & metabolism 23, 543–554, doi: 10.1016/j.beem.2009.05.008 (2009). 19945022

[b22] Ezzat S. & Asa S. L. Mechanisms of disease: The pathogenesis of pituitary tumors. Nature clinical practice. Endocrinology & metabolism 2, 220–230, doi: 10.1038/ncpendmet0159 (2006). 16932287

[b23] Asa S. L. & Ezzat S. The pathogenesis of pituitary tumors. Annual review of pathology 4, 97–126, doi: 10.1146/annurev.pathol.4.110807.092259 (2009). 19400692

[b24] Yacqub-Usman K., Richardson A., Duong C. V., Clayton R. N. & Farrell W. E. The pituitary tumour epigenome: aberrations and prospects for targeted therapy. Nature reviews. Endocrinology 8, 486–494, doi: 10.1038/nrendo.2012.54 (2012). 22525730

[b25] Zhou Y., Zhang X. & Klibanski A. Genetic and epigenetic mutations of tumor suppressive genes in sporadic pituitary adenoma. Mol Cell Endocrinol 386, 16–33, doi: 10.1016/j.mce.2013.09.006 (2014). 24035864 PMC3943596

[b26] Zhu X. *et al.* Deoxyribonucleic acid methyltransferase 3B promotes epigenetic silencing through histone 3 chromatin modifications in pituitary cells. The Journal of clinical endocrinology and metabolism 93, 3610–3617, doi: 10.1210/jc.2008-0578 (2008). 18544619

[b27] Ezzat S. Epigenetic control in pituitary tumors. Endocrine journal 55, 951–957 (2008). 18628575 10.1507/endocrj.k08e-082

[b28] Tateno T., Zhu X., Asa S. L. & Ezzat S. Chromatin remodeling and histone modifications in pituitary tumors. Mol Cell Endocrinol 326, 66–70, doi: 10.1016/j.mce.2009.12.028 (2010). 20060434

[b29] Farrell W. E. & Clayton R. N. Epigenetic change in pituitary tumorigenesis. Endocr Relat Cancer 10, 323–330 (2003). 12790793 10.1677/erc.0.0100323

[b30] Dudley K. J., Revill K., Clayton R. N. & Farrell W. E. Pituitary tumours: all silent on the epigenetics front. J Mol Endocrinol 42, 461–468, doi: JME-09-0009 [pii]10.1677/JME-09-0009 (2009). 19208779 10.1677/JME-09-0009

[b31] Asa S. L. & Ezzat S. Genomic approaches to problems in pituitary neoplasia. Endocr Pathol 25, 209–213, doi: 10.1007/s12022-013-9276-5 (2014). 24272682

[b32] Farrell W. E. Epigenetic mechanisms of tumorigenesis. Hormone and metabolic research = Hormon- und Stoffwechselforschung = Hormones et metabolisme 37, 361–368, doi: 10.1055/s-2005-870153 (2005). 16001328

[b33] Vandeva S. *et al.* The genetics of pituitary adenomas. Best practice & research. Clinical endocrinology & metabolism 24, 461–476, doi: 10.1016/j.beem.2010.03.001 (2010). 20833337

[b34] Ebrahimi A., Schittenhelm J., Honegger J. & Schluesener H. J. Histone acetylation patterns of typical and atypical pituitary adenomas indicate epigenetic shift of these tumours. J Neuroendocrinol 23, 525–530, doi: 10.1111/j.1365-2826.2011.02129.x (2011). 21453398

[b35] DeLellis R. A., Lloyd R. V., Heitz P. U. & Eng C. Pathology and genetics of tumours of endocrine organs. (IARC, 2004).

[b36] Farrell W. E. Epigenetics of pituitary tumours: an update. Current opinion in endocrinology, diabetes, and obesity 21, 299–305, doi: 10.1097/MED.0000000000000078 (2014). 24983395

[b37] Duong C. V. *et al.* Quantitative, genome-wide analysis of the DNA methylome in sporadic pituitary adenomas. Endocr Relat Cancer 19, 805–816, doi: 10.1530/ERC-12-0251 (2012). 23045325

[b38] Duong C. V., Yacqub-Usman K., Emes R. D., Clayton R. N. & Farrell W. E. The EFEMP1 gene: a frequent target for epigenetic silencing in multiple human pituitary adenoma subtypes. Neuroendocrinology 98, 200–211, doi: 10.1159/000355624 (2013). 24080855

[b39] Palmieri D. *et al.* Downregulation of HMGA-targeting microRNAs has a critical role in human pituitary tumorigenesis. Oncogene 31, 3857–3865, doi: 10.1038/onc.2011.557 (2012). 22139073

[b40] Kitchen M. O. *et al.* Epidrug mediated re-expression of miRNA targeting the HMGA transcripts in pituitary cells. Pituitary 18, 674–684, doi: 10.1007/s11102-014-0630-5 (2015). 25557289

[b41] Vila G. *et al.* Expression and function of sonic hedgehog pathway components in pituitary adenomas: evidence for a direct role in hormone secretion and cell proliferation. J Clin Endocrinol Metab 90, 6687–6694, doi: 10.1210/jc.2005-1014 (2005). 16159933

[b42] Ibáñez-Costa A. *et al.* In1-ghrelin splicing variant is overexpressed in pituitary adenomas and increases their aggressive features. Scientific reports 5 (2015). 10.1038/srep08714 PMC4649711 25737012

[b43] Hanahan D. & Weinberg R. A. The hallmarks of cancer. Cell 100, 57–70 (2000). 10647931 10.1016/s0092-8674(00)81683-9

[b44] Bracken A. P. *et al.* EZH2 is downstream of the pRB-E2F pathway, essential for proliferation and amplified in cancer. Embo J 22, 5323–5335, doi: 10.1093/emboj/cdg542 (2003). 14532106 PMC213796

[b45] Jacks T. *et al.* Effects of an Rb mutation in the mouse. Nature 359, 295–300, doi: 10.1038/359295a0 (1992). 1406933

[b46] Ezhkova E. *et al.* Ezh2 orchestrates gene expression for the stepwise differentiation of tissue-specific stem cells. Cell 136, 1122–1135, doi: 10.1016/j.cell.2008.12.043 (2009). 19303854 PMC2716120

[b47] Nakayama K. *et al.* Mice lacking p27(Kip1) display increased body size, multiple organ hyperplasia, retinal dysplasia, and pituitary tumors. Cell 85, 707–720 (1996). 8646779 10.1016/s0092-8674(00)81237-4

[b48] Franklin D. S. *et al.* CDK inhibitors p18(INK4c) and p27(Kip1) mediate two separate pathways to collaboratively suppress pituitary tumorigenesis. Genes Dev 12, 2899–2911 (1998). 9744866 10.1101/gad.12.18.2899 PMC317173

[b49] Bamberger C. M. *et al.* Reduced expression levels of the cell-cycle inhibitor p27Kip1 in human pituitary adenomas. Eur J Endocrinol 140, 250–255 (1999). 10216521 10.1530/eje.0.1400250

[b50] Lidhar K. *et al.* Low expression of the cell cycle inhibitor p27Kip1 in normal corticotroph cells, corticotroph tumors, and malignant pituitary tumors. J Clin Endocrinol Metab 84, 3823–3830 (1999). 10523037 10.1210/jcem.84.10.6066

[b51] Ougolkov A. V., Bilim V. N. & Billadeau D. D. Regulation of pancreatic tumor cell proliferation and chemoresistance by the histone methyltransferase enhancer of zeste homologue 2. Clinical cancer research : an official journal of the American Association for Cancer Research 14, 6790–6796, doi: 10.1158/1078-0432.CCR-08-1013 (2008). 18980972 PMC2690708

[b52] Schult D. *et al.* Expression pattern of neuronal intermediate filament alpha-internexin in anterior pituitary gland and related tumors. Pituitary, doi: 10.1007/s11102-014-0597-2 (2014). 25236435

[b53] Holsken A. *et al.* Adamantinomatous craniopharyngiomas express tumor stem cell markers in cells with activated Wnt signaling: further evidence for the existence of a tumor stem cell niche? Pituitary 17, 546–556, doi: 10.1007/s11102-013-0543-8 (2014). 24356780

[b54] Holsken A., Buchfelder M., Fahlbusch R., Blumcke I. & Buslei R. Tumour cell migration in adamantinomatous craniopharyngiomas is promoted by activated Wnt-signalling. Acta Neuropathol 119, 631–639, doi: 10.1007/s00401-010-0642-9 (2010). 20131060

[b55] Holsken A. *et al.* *Ex vivo* therapy of malignant melanomas transplanted into organotypic brain slice cultures using inhibitors of histone deacetylases. Acta Neuropathol 112, 205–215, doi: 10.1007/s00401-006-0082-8 (2006). 16773328

