# Supplementary material for: EZH2 is highly expressed in pituitary adenomas and associated with proliferation
Source: Sci Rep. 2015 Nov 23;5:16965. doi: 10.1038/srep16965 (PMC4655333; doi:10.1038/srep16965)
Supplement: Supplementary Information [file srep16965-s1.doc]

**EZH2 is highly expressed in pituitary adenomas and associated with proliferation**

David Schult^1,5^, Annett Hölsken^1,5^, Sonja Siegel^2,4^, Michael Buchfelder^2^, Rudolf Falhbusch^3^, Ilonka Kreitschmann-Andermahr^2,4^, Rolf Buslei^1*^

^1^Institute of Neuropathology, Friedrich-Alexander University Erlangen-Nürnberg (FAU), Schwabachanlage 6, 91054 Erlangen; ^2^Department of Neurosurgery, Friedrich-Alexander University Erlangen-Nürnberg (FAU), Schwabachanlage 6, 91054 Erlangen; ^3^Department of Neurosurgery, International Neuroscience Institute, Rudolf-Pichlmayr-Straße 4, 30625 Hannover; ^4^Department of Neurosurgery, University of Duisburg-Essen, Hufelandstraße 55, 45122 Essen;

^5^These authors contributed equally to this work;

**Supplemental data:**

The table contains all 184 examined tissue samples. Percentage of EZH2 and Ki-67 immunostaining was established by counting a minimum of 1000 cells. Tumor size refers on the largest diameter of the tumor stated in millimeter. In some cases the exact tumor size was not available retrospectively, however in that cases the size was stated as micro (<10 mm), macro (≥10 mm) or giant (≥30 mm). f = female, m = male. Controls = normal adenohypophyseal tissue, STH = somatotropin producing adenoma, PRL = prolactin producing adenoma, TSH = thyrotropin producing adenoma, FSH = follicle-stimulating hormone producing adenoma, LH = luteinizing hormone producing adenoma, ACTH = corticotropin producing adenoma, silent ACTH = clinically non-functioning ACTH producing adenoma.

| **EZH2 (%)** | **Ki-67 (%)** | **Size (mm)** | **Invasivity** | **Age (y)** | **Sex** |
| --- | --- | --- | --- | --- | --- |
| **Controls (n = 19)** | | | | | |
| 0.0 | 0.3 | - | - | 41 | f |
| 0.0 | 0.5 | - | - | 14 | f |
| 0.0 | 0.1 | - | - | 69 | m |
| 0.0 | 0.4 | - | - | 54 | m |
| 0.0 | 0.1 | - | - | 34 | f |
| 0.0 | 0.1 | - | - | 33 | f |
| 0.0 | 0.2 | - | - | 58 | f |
| 0.0 | 0.4 | - | - | 32 | m |
| 0.0 | 0.2 | - | - | 19 | f |
| 0.0 | 0.0 | - | - | 40 | m |
| 0.0 | 1.0 | - | - | 28 | f |
| 0.0 | 0.1 | - | - | 13 | m |
| 0.0 | 0.5 | - | - | 39 | m |
| 0.4 | 0.2 | - | - | 57 | m |
| 1.6 | 1.0 | - | - | 54 | f |
| 0.2 | 0.9 | - | - | 20 | f |
| 0.5 | 0.6 | - | - | 16 | m |
| 0.1 | 0.1 | - | - | 32 | f |
| 0.1 | 0.2 | - | - | 33 | f |
| **densely granulated STH (n = 14)** | | | | | |
| 0.2 | 0.7 | 5.5 | yes | 50 | m |
| 2.0 | 0.6 | 30 | yes | 50 | m |
| 1.7 | 1.2 | 13 | yes | 41 | f |
| 0.5 | 0.4 | 7 | no | 49 | m |
| 0.3 | 0.5 | 5 | yes | 50 | m |
| 1.8 | 2.1 | 22 | no | 65 | f |
| 0.8 | 0.8 | 10 | no | 76 | f |
| 0.4 | 0.3 | 20 | no | 41 | f |
| 0.1 | 0.2 | 18 | no | 47 | m |
| 1.7 | 1.4 | 9 | no | 48 | f |
| 3.3 | 3.1 | 15 | no | 30 | m |
| 1.3 | 1.5 | 30 | yes | 62 | m |
| 4.2 | 3.8 | 20 | yes | 34 | m |
| 1.9 | 2.4 | 19 | yes | 47 | m |
| **sparsely granulated STH (n = 10)** | | | | | |
| 2.1 | 1.3 | 35 | yes | 34 | f |
| 2.0 | 2.0 | 18 | no | 56 | f |
| 1.9 | 1.9 | 16 | no | 74 | m |
| 1.5 | 2.5 | 27 | no | 56 | f |
| 0.7 | 1.0 | 25 | yes | 32 | m |
| 1.2 | 1.1 | 44 | yes | 40 | m |
| 0.8 | 0.3 | 9 | no | 56 | f |
| 4.7 | 5.8 | 11 | no | 38 | f |
| 5.1 | 6.5 | 28 | yes | 54 | f |
| 2.0 | 2.4 | 10 | yes | 38 | m |
| **PRL (n = 13)** | | | | | |
| 1.4 | 3.4 | 23 | yes | 58 | m |
| 1.2 | 1.6 | 16 | no | 44 | f |
| 3.3 | 3.2 | 16 | no | 48 | m |
| 0.7 | 1.6 | 22 | no | 31 | m |
| 1.8 | 1.2 | giant | yes | 50 | f |
| 0.8 | 0.2 | 9 | no | 23 | f |
| 2.2 | 3.1 | 29 | yes | 37 | m |
| 0.5 | 0.7 | 15 | no | 37 | m |
| 1.3 | 1.1 | 6 | no | 30 | f |
| 1.3 | 0.9 | 7 | no | 45 | f |
| 3.5 | 6.5 | 6 | no | 24 | f |
| 3.8 | 4.2 | 14 | yes | 21 | f |
| 2.8 | 2.9 | 45 | yes | 35 | m |
| **mixed STH/PRL (n = 9)** | | | | | |
| 1.4 | 0.9 | 16 | no | 65 | f |
| 2.4 | 2.7 | 20 | yes | 37 | m |
| 2.8 | 2.9 | 13 | no | 57 | m |
| 1 | 1.6 | 30 | yes | 59 | f |
| 2.5 | 1.7 | 12 | no | 51 | f |
| 2.3 | 2.4 | 13 | no | 64 | f |
| 3.1 | 3.0 | 10 | no | 15 | f |
| 2.1 | 2.3 | 11 | no | 27 | m |
| 2.5 | 2.6 | 18 | yes | 37 | f |
| **Nelson’s Tumor (n = 7)** | | | | | |
| 0.6 | 1.5 | 19 | yes | 53 | m |
| 0.8 | 1.4 | 26 | yes | 66 | f |
| 13.0 | 7.5 | 36 | yes | 51 | m |
| 7.7 | 8.2 | 43 | yes | 75 | f |
| 7.0 | 7.4 | 11 | yes | 43 | f |
| 16.5 | 15.9 | 35 | yes | 55 | f |
| 2.5 | 3.0 | 38 | yes | 33 | f |
| **Null cell adenoma (n = 6)** | | | | | |
| 3.7 | 3.6 | 27 | yes | 23 | m |
| 0.8 | 0.7 | 22 | no | 57 | m |
| 1.1 | 1.0 | 15 | no | 46 | f |
| 4.6 | 5.0 | 21 | no | 54 | m |
| 6.2 | 6.5 | 38 | yes | 27 | m |
| 2.9 | 3.0 | 24 | yes | 52 | f |
| **TSH (n = 8)** | | | | | |
| 1.7 | 1.4 | 18 | no | 38 | f |
| 0.5 | 0.8 | 7-8 | no | 40 | m |
| 2.8 | 3.7 | 11 | no | 35 | f |
| 0.8 | 0.7 | 35 | yes | 35 | f |
| 2.6 | 2.8 | 18 | no | 48 | m |
| 8.0 | 9.0 | 10 | no | 23 | m |
| 1.0 | 2.8 | 17 | yes | 74 | f |
| 1.1 | 2.0 | 12 | no | 56 | f |
| **FSH (n = 11)** | | | | | |
| 0.2 | 1.0 | 31 | yes | 61 | m |
| 0.2 | 0.5 | 15 | no | 43 | f |
| 0.3 | 0.4 | 21 | no | 51 | f |
| 2.8 | 1.8 | 28 | yes | 61 | f |
| 1.2 | 1.9 | 35 | no | 61 | m |
| 3.1 | 2.8 | 44 | yes | 46 | m |
| 1.9 | 1.7 | 28 | no | 39 | m |
| 0.7 | 0.9 | 23 | yes | 73 | m |
| 0.8 | 1.2 | 37 | yes | 51 | m |
| 1.8 | 1.2 | 22 | no | 70 | m |
| 2.0 | 1.4 | 14 | yes | 51 | m |
| **LH (n = 13)** | | | | | |
| 1.0 | 1.3 | 39 | no | 71 | m |
| 2.2 | 3.3 | 43 | yes | 70 | m |
| 1.6 | 2.4 | 32 | no | 71 | f |
| 0.8 | 0.8 | 46 | yes | 68 | m |
| 1.5 | 2.8 | 33 | yes | 53 | f |
| 1.4 | 1.1 | 13 | no | 78 | m |
| 0.7 | 0.6 | 32 | no | 78 | m |
| 1.6 | 1.8 | 25 | no | 72 | f |
| 3.2 | 2.7 | 40 | yes | 71 | m |
| 2.3 | 2.2 | 19 | no | 63 | m |
| 2.2 | 2.5 | 14 | yes | 56 | m |
| 1.6 | 1.4 | 22 | no | 71 | m |
| 2.6 | 5.0 | 18 | no | 77 | m |
| **mixed FSH/LH (n = 39)** | | | | | |
| 1.2 | 1.5 | 14 | no | 57 | m |
| 0.5 | 0.8 | 30 | yes | 70 | f |
| 1.3 | 1.7 | 27 | no | 70 | f |
| 1.0 | 3.9 | 25 | no | 52 | m |
| 1.6 | 2.7 | 31 | yes | 64 | m |
| 2.1 | 1.7 | 33 | yes | 61 | m |
| 1.0 | 2.1 | 25 | no | 74 | m |
| 0.3 | 1.4 | 26 | yes | 88 | m |
| 2.8 | 2.3 | 17 | yes | 71 | m |
| 1.6 | 2.0 | 22 | no | 67 | m |
| 1.3 | 1.0 | 25 | no | 76 | f |
| 1.1 | 1.9 | 24 | yes | 44 | m |
| 1.9 | 2.2 | 22 | no | 75 | f |
| 0.9 | 1.4 | 24 | yes | 70 | m |
| 0.8 | 1.2 | 19 | no | 64 | m |
| 2.1 | 2.3 | 60 | yes | 59 | f |
| 1.6 | 1.3 | 15 | no | 56 | m |
| 2.8 | 3.6 | 41 | yes | 54 | m |
| 2.4 | 1.6 | 24 | no | 83 | m |
| 1.0 | 2.0 | 13 | no | 77 | m |
| 0.8 | 1.0 | 28 | no | 58 | m |
| 1.9 | 2.6 | 25 | yes | 73 | m |
| 0.7 | 1.4 | 25 | yes | 63 | m |
| 0.8 | 1.1 | 15 | no | 64 | f |
| 1.4 | 1.8 | 28 | yes | 49 | m |
| 1.5 | 1.7 | 57 | yes | 69 | m |
| 1.0 | 1.8 | 31 | no | 74 | m |
| 0.7 | 1.5 | 18 | no | 66 | f |
| 1.5 | 1.4 | 26 | yes | 65 | m |
| 1.0 | 0.6 | 12 | no | 61 | f |
| 1.1 | 0.8 | 18 | no | 74 | f |
| 2.6 | 3.2 | 25 | yes | 79 | m |
| 2.5 | 2.6 | 33 | yes | 55 | m |
| 3.0 | 2.8 | 24 | no | 57 | m |
| 2.8 | 2.5 | 40 | yes | 72 | m |
| 0.5 | 0.9 | 24 | no | 72 | m |
| 2.9 | 3.0 | 32 | yes | 55 | f |
| 2.1 | 2.1 | 17 | yes | 60 | f |
| 0.4 | 1.0 | 31 | yes | 47 | m |
| **ACTH (n = 13)** | | | | | |
| 0.2 | 0.3 | 2.5 | yes | 47 | f |
| 2.2 | 2.3 | 17 | yes | 53 | f |
| 0.6 | 1.1 | 3 | no | 37 | f |
| 1.6 | 1.6 | 30 | yes | 74 | f |
| 0.1 | 1.5 | 3 | no | 43 | m |
| 2.7 | 2.3 | 13 | no | 38 | f |
| 0.1 | 0.8 | 4 | no | 33 | f |
| 5.8 | 6.6 | 7.5 | no | 43 | f |
| 5.7 | 8.5 | 5 | no | 30 | m |
| 7.2 | 7.0 | 6 | no | 24 | f |
| 1.0 | 1.3 | 3 | no | 31 | f |
| 8.3 | 5.4 | 4 | no | 25 | f |
| 0.7 | 0.8 | 14 | yes | 51 | f |
| **silent ACTH (n = 10)** | | | | | |
| 0.6 | 0.9 | 38 | yes | 73 | m |
| 2.0 | 1.4 | 13 | no | 47 | m |
| 0.9 | 0.5 | 53 | yes | 71 | m |
| 1.0 | 0.7 | 35 | yes | 75 | m |
| 0.8 | 1.0 | 23 | yes | 56 | f |
| 2.0 | 2.2 | 14 | yes | 57 | m |
| 0.5 | 0.5 | 47 | yes | 40 | m |
| 1.8 | 1.1 | 12 | no | 60 | f |
| 2.3 | 2.5 | 14 | no | 36 | f |
| 6.0 | 1.4 | 40 | yes | 29 | m |
| **atypical adenoma (n = 10)** | | | | | |
| 3.5 | 5.8 | 25 | no | 37 | m |
| 3.8 | 4.2 | 23 | no | 41 | m |
| 5.5 | 4.8 | 32 | yes | 35 | f |
| 10.6 | 12.2 | 43 | yes | 45 | m |
| 8.1 | 9.1 | 41 | yes | 38 | m |
| 4.1 | 5.8 | 53 | yes | 20 | f |
| 17.4 | 11.3 | 43 | yes | 68 | f |
| 21.0 | 27.8 | 41 | yes | 84 | m |
| 17.6 | 19.8 | 30 | no | 35 | f |
| 7.7 | 10.4 | macro | yes | 65 | f |
| **Pituitary carcinoma (n = 2)** | | | | | |
| 25.0 | 6.6 | 38 | yes | 53 | m |
| 8.0 | 20.1 | 12 | no data | 57 | m |
